# Molecular Organ Lysate Imprinting: For Precision Recognition and Analysis of Organ‐Derived Proteins

**DOI:** 10.1002/rcm.10125

**Published:** 2025-08-27

**Authors:** Jatinderpal K. Sandhu, Donald J. L. Jones, Colleen B. Maxwell, Charlotte L. Gwinnell, Geraldine Williams, Thong Huy Cao, Paulene A. Quinn, Leong L. Ng

**Affiliations:** ^1^ Leicester van Geest MultiOMICs Facility University of Leicester Leicester UK; ^2^ Department of Cardiovascular Sciences University of Leicester Leicester UK; ^3^ National Institute for Health and Care Research Leicester Biomedical Research Centre University Hospitals of Leicester NHS Trust, Glenfield Hospital Leicester UK; ^4^ Leicester BHF Centre of Research Excellence Glenfield Hospital Leicester UK; ^5^ Leicester Cancer Research Centre, Leicester Royal Infirmary University of Leicester Leicester UK; ^6^ Department of Chemistry George Porter Building University of Leicester Leicester UK

**Keywords:** affinity ligands, dopamine polymers, mass spectrometry, molecular imprinting, organ lysate imprinting, plasma proteomics, protein capture, protein retrieval, targeted proteomics

## Abstract

**Rationale:**

Molecular imprinting has emerged as a promising strategy to create custom imprints for precision recognition of proteins. This study proposes using dopamine polymers as a novel approach to enhance the retrieval of proteins from human plasma. Dopamine polymers possess adhesive properties due to their ability to form hydrophobic interactions, π‐π, hydrogen bonding and van der Waals forces with various substrates; in this study, we have leveraged these adhesive properties to capture and retrieve proteins from complex biological samples.

**Methods:**

We imprinted proteins derived from mouse heart lysate and evaluated the performance of the resulting molecularly imprinted polymer for retrieval of the human protein cardiovascular disease plasma samples.

**Results:**

Our results demonstrated the retrieval of troponin T, fatty acid–binding protein, creatine kinase, lactate dehydrogenase, and myosin‐binding protein C. This novel application of dopamine polymers in protein enrichment and analysis facilitates the discovery of novel biomarkers from complex matrices, such as plasma, and promotes deeper insights into complex biological processes.

**Conclusions:**

This method, characterized by high specificity and stability, offers a new approach for the detection of low abundant proteins and provides a scientific basis for the future development of new diagnostic tools and personalized medical strategies.

## 1 Introduction

The use of plasma proteomics as a tool to profile proteins in clinical samples has gained traction in the literature recently, as approaches enabling the detection of several thousand proteins using liquid chromatography–mass spectrometry (LC–MS) in a high throughput manner have become more established and accessible [1, 2, 3]. Proteins constitute the most commonly utilized chemical group in diagnostic medicine [4], and blood plasma often represents the most accessible source of proteins for clinical analyses. It is minimally invasive to obtain and provides a molecular signature of each organ that it passes through thus reflects systemic changes associated with various diseases. Moreover, blood tests are already the most widely used test in diagnostic medicine [5]; they are performed across various clinical settings, including primary care facilities, hospitals, and even through self‐collection at home. Notably, blood plasma testing is cost‐effective, especially when juxtaposed with alternative techniques such as medical imaging [2].

Liquid chromatography coupled to mass spectrometry (LC–MS) is a sensitive analytical method, which is already widely used in the clinic for a number of small molecule assays in human plasma, including vitamin D [6], parathyroid hormone, and for determining adherence to antihypertensive and immunosuppressant drugs [7]. Profiling of proteins in blood using LC–MS could offer huge advantages in clinical medicine, including the discovery and clinical measurement of biomarkers for diagnosis, prognosis, and clinical management of disease. Proteomics may also provide targets for novel, personalized therapy by unravelling the molecular underpinning of disease.

There are, however, several challenges associated with translating these novel plasma biomarkers from research into a clinical setting. One of the main challenges is the dynamic range and complex nature of the plasma as an analytical matrix. Human plasma proteins span an extremely wide range of concentrations—far greater than the dynamic range of even the most cutting‐edge mass spectrometers. The concentration of proteins within plasma spans over 12 orders of magnitude, and up to 85% of plasma comprises just 5–10 very abundant proteins, in particular albumin, which makes up almost 55% of plasma, making plasma the most complex fluid for proteomics [8]. This introduces problems with detecting less abundant proteins in shotgun proteomics, with few very high abundance proteins masking the presence of thousands of lower abundance proteins that are valuable to discovery proteomics investigations in areas such as cardiovascular diseases. Various preanalytical strategies can address this challenge by reducing sample complexity before conducting LC–MS analysis. Typically, these strategies involve enriching low‐abundance proteins or depleting the most abundant proteins present in plasma.

Current enrichment methods include immunoaffinity chromatography, molecular weight gel‐based separation, size exclusion chromatography, stable isotope standards with capture by antipeptide antibodies (SISCAPA) [9], and filter‐aided enrichment. The isolation of extracellular vesicles (EVs) is also a method to enrich for low‐abundance proteins, but typical EV isolation techniques are costly and extremely low throughput. Fractionation of clinical samples can also be used to reduce the dynamic range, but again this significantly reduces throughput. Depletion methods such as albumin and IgG depletion, ProteoPrep Blue Albumin, and Multiple Affinity Removal Spin (MARS) cartridges enable the removal of the most abundant proteins, such as albumin, IgG, antitrypsin, IgA, transferrin, and haptoglobin, which improves the detection of lower abundant proteins. These methods are often expensive, labor‐intensive, can introduce batch effects, and are not practical for large clinical studies spanning thousands of samples [10]. Thus, substantial effort has been directed at improving enrichment and depletion strategies to access the full dynamic range of the human plasma proteome. To this end, molecular imprinting is emerging as a promising technique that can be utilized in a novel way as part of the proteomics workflow to achieve either enrichment or depletion.

Molecular imprinting has a wide range of applications across various fields, including sensor technology, fluorescence‐based assays, and sample purification such as solid‐phase extraction (SPE) [11, 12, 13], cancer biomarker detection, and chromatography [14]. There are multiple approaches for creating molecular imprints for specific applications, including surface and epitope imprinting, to name a few [15, 16, 17]. Typical molecular imprinting methods involve the incorporation of a template molecule, a functional monomer, a cross‐linker, and a polymerization initiator. This process produces a polymer matrix with recognition sites similar to the size, shape, and ionic/hydrophobic interactions of the template molecule. Subsequent removal of the template molecule leaves behind a cavity with a three‐dimensional structure, which can capture the analyte of interest [18].

Thus far, molecular imprinting has demonstrated ample success in the selectivity and recognition of small organic molecules and metal ions [19, 20] and offers an opportunity to create templates to specifically recognize endogenous proteins and peptides within biological samples. This approach offers many advantages compared to current sample enrichment and depletion techniques. These include enhanced specificity, stability, cost‐effectiveness, and time lag for the production of antibodies for immunocapture that are prone to batch‐to‐batch variation [21, 22].

**FIGURE 1:**
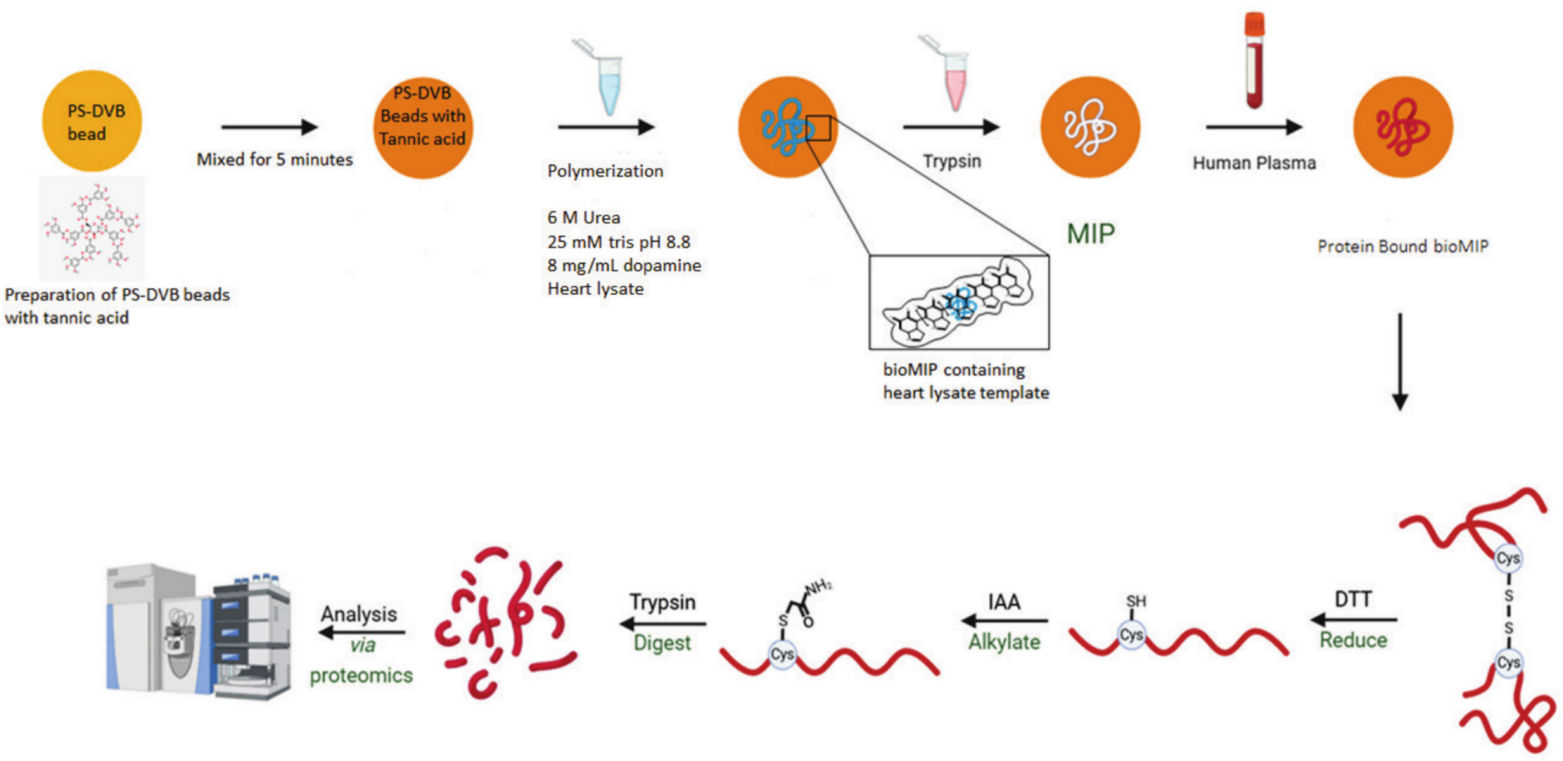
Workflow of the molecular imprinting technique utilizing a mouse heart lysate as a template to create an imprinted polymer (bioMIP) designed for heart‐related proteins. Polymerization onto polystyrene beads occurred in the presence of mouse heart lysate, dopamine as the monomer, tris, and urea. The mouse heart lysate, which was the template, was removed after the polymerization step, leaving behind cavities that were complementary to heart‐related ligands (polystyrene bioMIP). The polystyrene bioMIP was evaluated by contacting the cavities that contained the heart‐related ligands with five plasma samples from myocardial infarction patients. The polystyrene bioMIP was then reduced/alkylated and trypsinized overnight, and the samples were analyzed using a parallel reaction monitoring (PRM)–targeted approach.

In this paper, we describe a novel process of synthesizing and utilizing bioinspired molecularly imprinted polymers (bioMIPs) as a means of identifying a number of proteins associated with myocardial infarction from plasma. A bioMIP is a biologically molecularly imprinted polymer that is designed to mimic biological recognition processes. This was achieved by polymerizing dopamine in the presence of polystyrene beads and urea via the workflow shown in Figure 1. In a novel approach, the template employed to generate these molecular imprints was derived from a mouse heart lysate. The imprint created in the presence of mouse heart lysate was called a bioinspired molecular imprint (bioMIP) compared to a non‐imprint where buffer was used instead and termed non‐imprinted polymer (NIP).

In this study, we focus on the enrichment of proteins analyzed using an LC–MS bottom‐up proteomics approach. Thus, it was important to ensure the molecular imprinting process remained compatible with downstream mass spectrometry analysis. To achieve this, it was necessary to select a mass spectrometry‐friendly monomer, such as dopamine. Dopamine undergoes polymerization through oxidation in ambient air [23]. Using harsh chemicals would lead to further rigorous wash steps; characterization of these wash steps would need to take place to be certain that there is no residue left of these chemicals. The rapid polymerization process eliminates the requirement for intermediates to initiate polymerization and takes place even in the presence of urea. The presence of urea is significant in proteomics because it denatures proteins, facilitating the dissociation of loosely bound proteins, therefore mitigating downstream issues related to the albuminome [10]. In a novel approach, the template employed to generate the molecular imprints in this work was derived from a mouse heart. The removal of this template by trypsinization ensures no carryover of mouse proteins to subsequent stages. During heart injury, there is a release of proteins from the heart tissue into blood plasma that can be used by cardiologists to diagnose disease and direct downstream management.

A heart attack, or myocardial infarction, typically occurs when there is a blockage in the heart arteries leading to a lack of oxygen reaching the heart muscle. This is usually as a result of coronary artery disease, where plaque has built up in the arteries in response to a variety of risk factors including smoking, high blood pressure, high cholesterol, age, and genetic factors. Patients presenting at hospital accident and emergency departments with chest pain and suspected myocardial infarction account for 5% of admissions (2017–2018). However, myocardial infarction only occurs in 20% of these admissions [24]. Blood tests for cardiac biomarkers can quickly test whether the patient is having a myocardial infarction or is at risk for future events, reducing unnecessary hospital admissions and downstream investigations with more expensive imaging tests such as computed tomography (CT). During the last 20 years, highly sensitive and specific methods for the detection of myocardial infarction have been developed. These include troponin I, troponin T and C, creatinine kinase (M), L‐lactate dehydrogenase (A and B chain), fatty acid–binding protein (FABPH), and myoglobin. These biomarkers have been incorporated into recognized diagnostic criteria for acute myocardial infarction by the National Institute for Health and Care Excellence [25, 26]. We therefore incorporated a number of these biomarkers into a putative panel used to diagnose myocardial infarction: troponin T (TNNT2), fatty acid‐binding protein (FABPH), creatine kinase M type (KCRM), L‐lactate dehydrogenase (A and B chain), and cardiac myosin‐binding protein C3 (MYPC3) [26]. One limitation of this study is the potential variability introduced by different batches of lysate, which may result in varying types and degrees of molecular imprinting. Studies should therefore preferably be performed with the same batch of lysate.

## 2 Experimental Section

### 2.1 Chemicals and Materials

All solvents (optima grade) were obtained from Sigma‐Aldrich (Poole, Dorset, UK) or Thermo Fisher Scientific (Loughborough, Leicester, UK). Waters total recovery vials were purchased from Waters (Elstree, Hertfordshire, UK). Easy‐spray columns and cartridge trap columns were obtained from Thermo Scientific (Loughborough, Leicester, UK). Urea, poly(styrene‐co‐divinylbenezene) (PS‐DVB, CAS No: 9003‐70‐7) 8‐μm particle size, tannic acid (CAS No: 1401‐55‐4), dopamine hydrochloride (CAS No: 62‐31‐7), and phosphate buffered saline (PBS) tablets (PBS, pH 7.4, CAT No: P4417‐100TAB) were purchased from Sigma‐Aldrich (Dorset, UK). Tris (hydroxymethyl)aminomethane (CAS No: 77‐86‐1) was purchased from Thermo Scientific (Loughborough, Leicester, UK).

### 2.2 Preparation of Substrate to Be Used in Molecular Imprinting

PS‐DVB (1 g, 8‐μm particle size, 1 mL) was added to tannic acid (0.5 g) and mixed with water (10 mL) for 5 min. Tannic acid improved the adsorption of proteins to the polystyrene. PS‐DVB was coated with tannic acid [27] to prevent the polystyrene from floating in solution and therefore easier to work with.

### 2.3 bioMIP Preparation Process

A solution containing dopamine hydrochloride (8 mg/mL, 1 mL), water (2 mL), tris buffer (200 mM, 200 μL), urea (6 M), and heart supernatant (40 mg/mL, 25 μL, native) was added to the template molecular imprint PS‐DVB (1 mL, approximate concentration of 0.1 g/mL). This mixture was incubated on a rotor for 72 h. After incubation, PS‐DVB beads were centrifuged at 3000 rpm for 10 min, and the supernatant was removed. The NIP was produced concurrently to the bioMIP, with the addition of heart lysate and urea replaced with 2 mL of water.

### 2.4 Preparation of Template Proteins Derived From Mouse Heart Tissue Proteins in Urea

Mouse heart tissue (0.5 mg) was homogenized using bead bug micro tube centrifuge zirconium beads (1.5 μM) (Model D2400, Benchmark Scientific Inc), with PBS (1 tablet per 200 mL of distilled water)/ethylenediaminetetraacetic acid (EDTA) (1 M, 2 μL). A bicinchoninic acid assay was carried out for protein concentration.

### 2.5 Template Removal

bioMIP PS‐DVB bead underwent a 3% acetic acid/20% isopropanol, 1 mL, wash, repeated three times, followed by a sodium chloride (NaCl) wash (0.5 M, 1 mL). Each time the mixture was centrifuged (3000 rpm, 10 min) and supernatant was removed. Ethanolamine (100 mM, 5 mL) was added to the bioMIP and incubated for 24 h on a rotor. The supernatant was centrifuged (3000 rpm, 10 min) and removed; the pellet was washed with ammonium bicarbonate (1 mL, 50 mM, pH 8.5). The mixture was centrifuged (15 000 rpm, 10 min) and supernatant was discarded. The bioMIP cavities containing the template protein were trypsinized overnight to remove any mouse proteins. The Molecularly Imprinted Polymer (MIP) PS‐DVB was designed using a mouse heart lysate as a template. After thorough cleaning to remove all mouse proteins, the resulting cavities became complementary to all heart proteins, reflecting the original imprinting process. To demonstrate proof of concept using both a mouse heart lysate as the template and human plasma as the recontact enrichment sample, we selected the following proteins: TNNT2, FABPH, KCRM, L‐lactate dehydrogenase (A and B chains), and MYPC3. While any heart‐related proteins could have been chosen, we specifically selected these because of access to cardiovascular patient samples from individuals diagnosed with myocardial infarction (MI).

### 2.6 Procedure of Biological Sample Preparation Using the bioMIP

Human plasma (50 μL, 300 μL, urea 6 M) was added to the bioMIP PS‐DVB (1 mL) and left on a rotor for 1 h. The mixture was centrifuged (1500 rpm, 10 min) and the supernatant was removed, and the pellet was washed with ammonium bicarbonate (1 mL, 50 mM, pH 8.5). The mixture was centrifuged (15 000 rpm, 10 min) and the supernatant was discarded. The bead was acidified with formic acid (1%) to elute proteins and centrifuged (15 000 rpm, 10 min). The bead supernatant was reduced, alkylated, trypsinized, and desalted using SPE. The sample was freeze dried overnight, reconstituted in formic acid (0.1%, 20 μL). The sample was analyzed using mass spectrometry (2 μL injected on column).

### 2.7 Patient Samples

Five patients with myocardial infarction in this study were selected from the Leicester Acute Myocardial Infarction Peptide study (LAMP) [28]. Nine hundred eighty patients were admitted into the coronary care unit (CCU) at the Leicester Royal Infirmary; these patients were recruited consecutively with acute myocardial infarction (718 men, median [range] age 66 [24–95] years), with follow‐up over 342 (range 0–764) days. AMI was diagnosed if a patient had chest pain lasting >20 min, diagnostic serial ECG changes consisting of new pathological Q waves or ST‐segment and T‐wave changes, and a plasma KCRM elevation greater than twice normal or cardiac troponin I level > 0.1 ng/mL. AMI was subcategorized into ST‐segment elevation myocardial infarction (STEMI) or non‐STEMI. Exclusion criteria were known malignancy or surgery in the previous month [28].

### 2.8 Instrumentation for Mass Spectrometry Analysis

Tryptic peptides were separated on an Ultimate 3000 RSLC Nano HPLC system (Dionex/Thermo Fisher Scientific, Bremen, Germany). Samples were loaded onto a cartridge‐based trap column, using a 300 μm × 5 mm C18 PepMap (5 μm, 100 Å) and then separated using an Easy‐Spray PepMap C18 column (75 μm × 50 cm), with a gradient from 3% to 10% B in 10 min, 10%–50% B in 37 min, 50%–90% in 9 min, and 90%–3% in 26 min, where mobile phase A was 0.1% formic acid in water and mobile phase B, 80%/20% acetonitrile/water in 0.1% formic acid. Flow rate was 0.3 μL/min. The column was operated at a constant temperature of 40°C.

NanoHPLC system was coupled to a Q‐Exactive mass spectrometer (ThermoScientific, Bremen, Germany). The Q‐Exactive was operated in the data‐dependent top 10 mode; full MS scans were acquired at a resolution of 70 000 at *m/z* 200–2000, with an ACG (ion target value) target of 1e6, maximum fill time of 50 ms. MS2 scans were acquired at a resolution of 17 500 with an ACG target of 1e5 and maximum fill time of 100 ms. The dynamic exclusion was set at 30.0 s to prevent repeat sequencing of peptides.

### 2.9 Skyline and PRM

Skyline (MacCoss lab software, (64‐bit) version 24.1.0.199) [29] was used to create a list of peptides, typically 1 peptide per protein and 1 or 2 transitions per peptide. This inclusion list with scheduling times to target, TNNT2, FABPH, KCRM, L‐lactate dehydrogenase (A and B chain), and MYPC3 was imported into a PRM MS method. Five patient samples with NTproBNP levels above 3000 pmol/L were analyzed using this targeted approach.

To proceed with this approach, there is a need for a spectral library. This is acquired in DDA top 10 mode; full MS scans were acquired at a resolution of 70 000 at 200 *m/z*, scanning from 50 *m/z* to 2000 *m*/*z*. An automatic gain control (AGC) of 1e6 and a maximum fill time of 50 ms were used. MS2 scans were acquired at a resolution of 17 500, with an AGC target of 1e5 and a maximum fill time of 100 ms. The dynamic exclusion was set at 30 s. This raw data file was processed in PD 2.4, using Sequest HT against a Uniprot human fasta file, precursor mass tolerance 10 ppm, and 1 missed cleavage. The following modifications were used: carbamidomethylation of cysteine as a static modification and methionine oxidation as a variable modification. All data analysis was filtered using a 1% false discovery rate (FDR). This data was imported into Skyline [29] using the following criteria to filter the peptides: peptides between 8–25 residues in length, no missed cleavages, and unique peptides, which were selected; these peptides were further filtered depending on their retention time on the chromatographic profile, peak intensity, and a charge state of either 2 or 3. These peptides were used to create an inclusion list, and their scheduled monitoring times were exported into Xcalibur (ThermoScientific, UK) (Table 1).

**TABLE 1:** Targeted PRM protein, peptide, and precursor.

Protein	Peptide	Precursor ion M/Z	Product ion M/Z
		Precursor ion charge 2+	Product ion charge 1+
Troponin T	VDFDDIHR	508.7434	540.2888
Myosin‐binding protein C3	EPVFIPRPGITYEPPNYK	1059.057	618.3245 586.3235 689.3828
Fatty acid–binding protein	SIVTLDGGK	445.2531	489.2667, 629.3504 689.3828
L‐lactate dehydrogenase A‐chain	DLADELALVDVIEDK	829.4302	940.499 1055.525
Creatine kinase M‐type	SFLVWVNEEDHLR	822.4125	1197.5647
L‐lactate dehydrogenase B‐chain	IHPVSTMVK	506.2864	565.3014

## 3 Results and Discussion

The tryptic peptides of these diagnostic proteins were analyzed using a targeted LC–MS parallel reaction monitoring (PRM) approach. Skyline [29] was used to generate a PRM isolation list targeting peptides unique to each of the myocardial infarction–related *Homo sapiens* proteins: TNNT2, FABPH, KCRM, L‐lactate dehydrogenase (A and B chain), and MYPC3. A basic local alignment search tool (BLAST) [30] was carried out and showed the homology between mouse and human in Table 2. This highlights that there is a strong homology between *Mus musculus* and *Homo sapiens* heart proteins and indicates the potential of using mouse heart lysate as a molecular imprint template to capture low‐abundance proteins from human plasma. The bioMIPs were developed using mouse heart as described in the workflow in Figure 1 and used to isolate proteins from the plasma of five donors.

**TABLE 2:** The protein homology of TNNT2, FABPH, KCRM, L‐lactate dehydrogenase (A and B chain), and MYPC3 (%) between *Mus musculus* and *Homo sapiens* .

Homology of *Mus musculus* with *Homo sapiens* protein (%)					
Troponin T	Myosin‐binding protein C,	Creatine kinase M‐type	L‐lactate dehydrogenase A chain	L‐lactate dehydrogenase B chain	Fatty acid–binding protein
93	89	97	94	98	86

The plasma samples from five patients with NTproBNP levels exceeding 3000 pmol/L and one control with no indication of myocardial infarction were obtained. These patients were participants in the Leicester Acute Myocardial Infarction Peptide (LAMP) study [28]. The plasma samples were contacted with the heart bioMIP as described in Figure 1 and the PRM method described above subsequently used to measure the biomarker panel. Results are shown in Table 3. The bioMIP shows that most of the disease clinical samples successfully captured the myocardial infarction proteins. The control sample derived from plasma of healthy patient with no history of myocardial infarction indicated that the NIP did not detect any of the myocardial infarction–related proteins, while the bioMIP detected L‐lactate dehydrogenase A chain, MYPC3, and FABPH. Our objective in this paper focused on demonstrating a proof‐of‐concept rather than comparing a quantitative approach to disease versus control samples. Thus, we created a bioMIP using mouse heart proteins and successfully captured six biomarker proteins in human myocardial infarction patient samples. The NIP served as the control.

**TABLE 3:** Five clinical samples (*n* = 1 each) from individuals diagnosed with myocardial infarction and one control individual with no known diagnosed myocardial infarction were analyzed using bioMIP and NIP. The bioMIP after trypsinization without human plasma was also analyzed. NIP samples overall exhibited no selective capture of the six target proteins. The results represent peak area under the curve (data has been logged) for peptides associated with the respective protein of interest specifically targeted in a PRM method. Patients generally showed the presence of TNNT2, FABPH, KCRM, L‐lactate dehydrogenase (A and B chain), and MYPC3.

Imprint	Sample	L‐lactate dehydrogenase A chain (Log_10_ peak area)	Troponin T (Log_10_ peak area)	Creatine kinase M‐type (Log_10_ peak area)	L‐lactate dehydrogenase B chain (Log_10_ peak area)	Myosin‐binding protein C, (Log_10_ peak area)	Fatty acid–binding protein (Log_10_ peak area)
bioMIP	Mouse	**0**	**0**	**0**	**0**	**0**	**0**
bioMIP	Plasma control	**287 250**	**0**	**0**	**0**	**106 779** **(IF = 43.8)**	**1295**
NIP	Plasma control	**0**	**0**	**0**	**0**	**2438**	**0**
bioMIP	Patient A	**1 047 521**	**48 681**	**565 584**	**0**	**99 632**	**88 760**
NIP	Patient A	**0**	**0**	**0**	**0**	**0**	**0**
bioMIP	Patient B	**1 461 129**	**7 664 329 (IF = 21.6)**	**1 272 436**	**302 435**	**91 086**	**5647**
NIP	Patient B	**0**	**354 455**	**0**	**0**	**0**	**0**
bioMIP	Patient C	**210 963**	**106 047** **(IF = 0.78)**	**380 569**	**144 860**	**502 114**	**57 868**
NIP	Patient C	**0**	**135 974**	**0**	**0**	**0**	**0**
bioMIP	Patient D	**11 438**	**142 929** **(IF = 0.26)**	**1 072 634**	**272 013**	**0**	**417 258**
NIP	Patient D	**0**	**549 270**	**0**	**0**	**0**	**0**
bioMIP	Patient E	**369 718**	**552 364**	**588 243**	**53 524**	**54 462**	**18 403**
NIP	Patient E	**0**	**0**	**0**	**0**	**0**	**0**

Table 3 includes the selectivity factor of a bioMIP, and this is determined by expressing bioMIP and NIP as an imprinting factor (IF). If this factor is greater than 1.0 that indicates enrichment, the IF for most cases is infinity due to the zero reading in the NIP. IF is measured from the following equation: IF = Binding on bioMIP/Binding on NIP, where bioMIP and NIP are the molecularly imprinted polymer and non‐imprinted polymer, respectively. The IF has been calculated for proteins where the NIP has shown nonspecific binding. The IF for troponin indicates that the protein may not exhibit strong selectivity. However, the IF for some cases is infinity due to the zero reading of the NIP.

There are a number of applications for MIPs, which currently exist; these rely on the enrichment or depletion of small molecules, drugs, and sensors. To our knowledge, this paper describes for the first time a novel method for creating bioMIPs to a collection of large biomacromolecules within mouse heart for application in plasma proteomics. There have been a number of issues to date regarding the challenges of creating bioMIPs in the field of proteomics; these include steric hindrance during template removal, the general complexity of proteins, and the use of nonmass spectrometry‐compatible chemicals [31]. Our method describes the use of surface imprinting with polystyrene and dopamine as a monomer, both of which are mass spectrometry‐compatible and do not require harsh chemicals.

**FIGURE 2:**
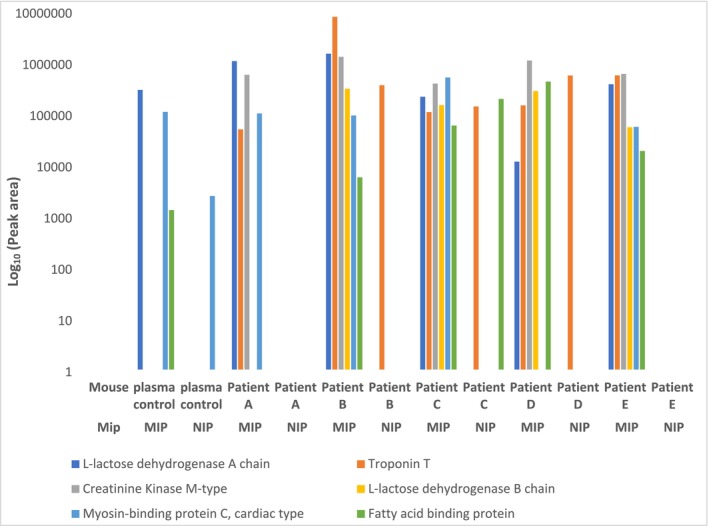
Targeted mass spectrometry data specifically detected imprints of muscle‐related heart proteins, including troponin T, L‐lactate dehydrogenase A and B chain, creatine kinase M type, myosin‐binding protein C cardiac type, and FABPH. The log_10_(peak area) is shown for each target protein.

The six proteins selected to be measured in this study are known markers of myocardial infarction and are examples of low concentration proteins measured by MS. Our novel proof‐of‐concept approach successfully created molecular imprints of protein extracts from whole mouse heart to enrich heart‐related proteins from human plasma. We have specifically achieved imprints to the structural and muscle‐related heart proteins TNNT2, FABPH, KCRM, L‐lactate dehydrogenase (A and B chain), and MYPC3 as shown in Table 2 and Figure 2. These proteins were successfully enriched from plasma through the organ lysate molecular imprinting approach, which are absent in the non‐imprinted polymers, thus removing the possibility of simple adsorption or nonspecific binding being the mechanism of capture. The results show that in a small number of myocardial infarction patients, the presence of multiple cardiac biomarkers can be detected. Interestingly, these biomarkers have differing profiles, but this may well reflect the different disease pathology in what is a small number of patients. A small number of peak areas corresponding to TNNT2 were observed in the non‐imprinted polymer samples from patient C and D. This likely results from nonspecific binding, indicating that the bioMIP may not exhibit strong selectivity or effectiveness for this particular protein in these cases.

## 4 Conclusions

Future studies will look to provide a quantitative approach through the incorporation of isotopically labelled stable isotopes or nonhuman surrogate internal standards [32], which will enable precise determination of the disease burden in the patient. In this proof‐of‐concept study, we used polystyrene to bind to the polydopamine. However, by carrying out the binding to magnetic or glass beads, we could eliminate the use of organic monomers, which would be preferable from an environmental perspective. One limitation of this approach is that it relies on high protein homology between mice and humans. There will be incidences where low homology would result in low capture affinity/avidity for the binding site. To address the challenges posed by low‐homology proteins, during the molecular imprinting process, designing synthetic receptors or imprinted polymers based on the specific sequence and structure of the human protein, rather than relying on mouse homologs, could improve specificity and binding efficiency. Using organs from species with a closer homology to humans is a possibility, or using human biopsy tissue obtained with consent. Other imprinting approaches using human‐derived cell lines from tissues, phospholipids [33] or glycans [34] of interest can be used, which do not rely on human and mouse homology. However, in this study, the six chosen proteins had in excess of 85% homology between mouse and human. The sample size is small, and future work would include increasing this sample size to improve the statistical significance of this study and the reliability of the results. Increasing the sample size would also look into concentration ranges and use a more targeted approach utilizing a triple quad mass spectrometer, which would provide linear ranges. This would also include the generation of a standard curve using known protein concentrations using protein standards such as bovine serum albumin. This would ensure the assay is quantitative. The generation of calibration lines could use protein standards such as bovine serum albumin. The analysis of patient samples was a single set of experiments that provide a proof‐of‐concept; future work would include optimization and validation of the imprinting process. Initial protocol development included varying concentrations of dopamine, tris, protein concentration, and time of polymerization using albumin as an experimental target prior to experiments on organ lysate imprinting [35].

The use of whole‐organ lysate bioMIPs can potentially aid new therapies. Akin to standard MIPs, which can be used to assess the most important epitopes [13, 14, 15], whole‐organ lysate bioMIPs could potentially be used in a similar way by identifying the peptides at the capture site. Creating whole‐organ lysate template bioMIPs can also be used for existing biomarker detection, particularly in cases where analysis without enrichment is challenging because of low protein abundance, and early monitoring of disease progress in personalized medicine. Thus, we have shown a proof‐of‐concept affirming the efficacy of our approach in enriching heart proteins from human plasma samples. Our method successfully detected key myocardial infarction biomarkers, including TNNT2, FABPH, KCRM, L‐lactate dehydrogenase (A and B chain), and MYPC3.

Future work utilizing this method opens an exciting area for the use of bioMIPs in proteomics cardiovascular biomarkers using mass spectrometry.

## Author Contributions

**Jatinderpal K Sandhu:** Conceptualization, Investigation, Writing –original draft, Methodology, Validation, Writing – review and editing, Software. **Donald JL Jones:** Funding acquisition, Writing – review and editing, Supervision. **Colleen B Maxwell:** Writing – review and editing, Data curation. **Charlotte L Gwinnell:** Visualization. **Geraldine Williams:** Writing – review and editing. **Thong Huy Cao:** Writing – review and editing, Data curation. **Paulene Quinn:** Writing – review and editing. **Leong L Ng:** Funding acquisition, Methodology, Writing – review and editing, Supervision.

## Ethics Statement

The study complied with the Declaration of Helsinki and was approved by the local ethics committee; written informed consent was obtained from patients.

## Conflicts of Interest

The authors declare no conflicts to declare.

## Data Availability

The data that support the findings of this study are available from the corresponding author upon reasonable request.
